# Poly[[diaqua­bis­[μ_4_-5-nitro­isophthalato-κ^4^ *O* ^1^:*O* ^1^:*O* ^3^:*O* ^3′^]bis­[μ_3_-pyridine-4-carboxyl­ato-κ^3^ *O*:*O*′:*N*]tricobalt(II)] tetra­hydrate]

**DOI:** 10.1107/S1600536812011269

**Published:** 2012-03-21

**Authors:** Xia Yin, Jun Fan, Jingling Xin, Shengrun Zheng, Weiguang Zhang

**Affiliations:** a School of Chemistry and Environment, South China Normal University, Guangzhou 510006, People’s Republic of China

## Abstract

The title compound, {[Co_3_(C_6_H_4_NO_2_)_2_(C_8_H_3_NO_6_)_2_(H_2_O)_2_]·4H_2_O}_*n*_, exhibits a two-dimensional layer-like structure in which the Co^II^ ions exhibit two kinds of coordination geometries. One nearly octa­hedral Co^II^ ion with crystallographic inversion symmetry is coordinated to six carboxyl­ate O atoms from four bridging 5-nitro­isophthalate (NIPH) ligands and two isonicotinate (IN) anions, while the other type of Co^II^ ion binds with one N atom and one carboxyl­ate O atom from two IN anions, two carboxyl­ate O atoms from two different NIPH anions and one ligated water mol­ecule, displaying a distorted square-pyramidal coordination geometry. Three adjacent Co^II^ ions are bridged by six carboxyl­ate groups from four NIPH ligands and two IN anions to form a linear trinuclear secondary building unit (SBU). Every trinuclear SBU is linked to its nearest neighbours in the *ab* plane, resulting in a two-dimensional layer-like structure perpendicular to the *c* axis. Along the *a*-axis direction neighbouring mol­ecules are connected through carboxyl­ate and pyridyl units of the IN anions, along the *b* axis through carboxyl­ate groups of the NIPH ligands. The H atoms of one free water mol­ecule are disordered in the crystal in a 1:1 ratio. Typical O—H⋯O hydrogen bonds are observed in the lattice, which include the following contacts: (*a*) between coordinated water mol­ecules and carboxyl­ate O atoms of the NIPH anions, (*b*) between lattice water mol­ecules and carboxyl­ate O atoms of the NIPH anions, and (*c*) between coordinated and lattice water mol­ecules. These inter­molecular hydrogen bonds connect the two-dimensional layers to form a three-dimensional supra­molecular structure.

## Related literature

For general background to the design and synthesis of coordination polymers, see: Jiang *et al.* (2010 ▶); Ma *et al.* (2009 ▶); Natarajan & Mahata (2009 ▶); Zang *et al.* (2006 ▶). For complexes with isonicotinate, see: Amo-Ochoa *et al.* (2010 ▶). For complexes with 5-nitro­isophthalate, see: Chen *et al.* (2006 ▶, 2010 ▶); Sun *et al.* (2010 ▶). For related compounds, see: Du *et al.* (2008 ▶); Luo *et al.* (2003 ▶); Wang *et al.* (2009 ▶). 
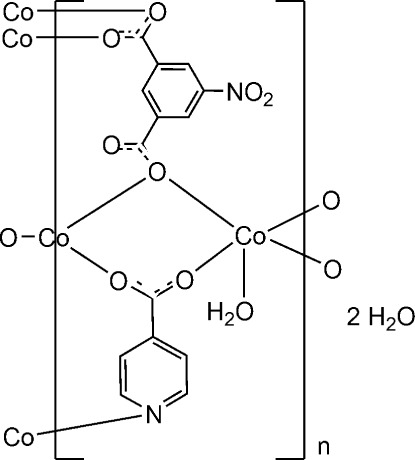


## Experimental

### 

#### Crystal data

[Co_3_(C_6_H_4_NO_2_)_2_(C_8_H_3_NO_6_)_2_(H_2_O)_2_]·4H_2_O
*M* *_r_* = 947.32
Triclinic, 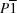

*a* = 9.1890 (18) Å
*b* = 9.3548 (19) Å
*c* = 10.390 (2) Å
α = 78.74 (3)°
β = 88.64 (3)°
γ = 73.68 (3)°
*V* = 840.2 (3) Å^3^
*Z* = 1
Mo *K*α radiation
μ = 1.57 mm^−1^
*T* = 298 K
0.35 × 0.28 × 0.16 mm

#### Data collection

Bruker SMART APEXII CCD area-detector diffractometer
Absorption correction: multi-scan (*SADABS*; Bruker, 2002 ▶) *T*_min_ = 0.610, *T*_max_ = 0.788
4357 measured reflections
2976 independent reflections
2667 reflections with *I* > 2σ(*I*)
*R*_int_ = 0.012

#### Refinement

*R*[*F* ^2^ > 2σ(*F* ^2^)] = 0.027
*wR*(*F* ^2^) = 0.071
*S* = 1.02
2976 reflections
271 parameters
10 restraints
H atoms treated by a mixture of independent and constrained refinement
Δρ_max_ = 0.40 e Å^−3^
Δρ_min_ = −0.40 e Å^−3^

### 

Data collection: *APEX2* (Bruker, 2007 ▶); cell refinement: *APEX2*; data reduction: *SAINT* (Bruker, 2007 ▶); program(s) used to solve structure: *SHELXS97* (Sheldrick, 2008 ▶); program(s) used to refine structure: *SHELXL97* (Sheldrick, 2008 ▶); molecular graphics: *SHELXTL* (Sheldrick, 2008 ▶); software used to prepare material for publication: *SHELXTL*.

## Supplementary Material

Crystal structure: contains datablock(s) I, global. DOI: 10.1107/S1600536812011269/zl2461sup1.cif

Structure factors: contains datablock(s) I. DOI: 10.1107/S1600536812011269/zl2461Isup2.hkl

Additional supplementary materials:  crystallographic information; 3D view; checkCIF report

## Figures and Tables

**Table 1:** Hydrogen-bond geometry (Å, °)

*D*—H⋯*A*	*D*—H	H⋯*A*	*D*⋯*A*	*D*—H⋯*A*
O1*W*—H1*W*⋯O3^i^	0.84	2.05	2.861 (3)	160
O1*W*—H2*W*⋯O2*W*^ii^	0.85	1.93	2.773 (3)	173
O3*W*—H5*W*⋯O2^iii^	0.82	2.01	2.820 (2)	168
O3*W*—H6*W*⋯O1*W*^iv^	0.83	1.83	2.648 (3)	167
O2*W*—H4*WA*⋯O2^v^	0.85 (2)	2.27 (2)	3.098 (4)	165 (8)
O2*W*—H3*WA*⋯O6^vi^	0.83 (2)	2.38 (6)	3.055 (3)	138 (8)
O2*W*—H3*WB*⋯O2*W*^ii^	0.87 (2)	2.49 (3)	3.297 (6)	154 (6)
O2*W*—H4*WB*⋯O6^vi^	0.89 (5)	2.54 (5)	3.055 (4)	118 (3)
O2*W*—H4*WB*⋯O2^v^	0.89 (5)	2.54 (5)	3.098 (3)	121 (4)

Symmetry codes: (i) 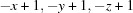; (ii) 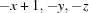; (iii) 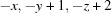; (iv) 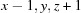; (v) 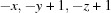; (vi) 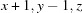.

**Fig. 1.**
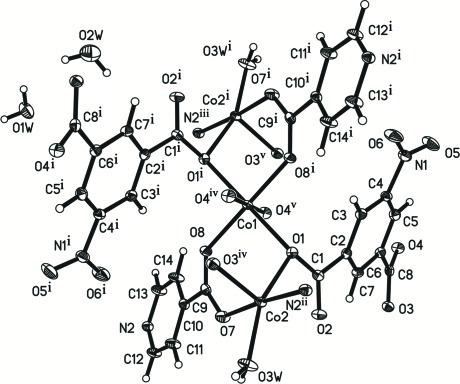
The molecular structure of the title compound, with displacement ellipsoids drawn at the 30% probability level. All H atoms are drawn as spheres of arbitrary radii. [Symmetry codes: (i): -x, -y + 1, -z + 1; (ii): x - 1, y, z; (iii): -x + 1, -y + 1, -z + 1; (iv): x, y - 1, z; (v): -x, -y + 2, -z - 1]. Disorder of H atoms of O2W is omitted for clarity.

**Fig. 2.**
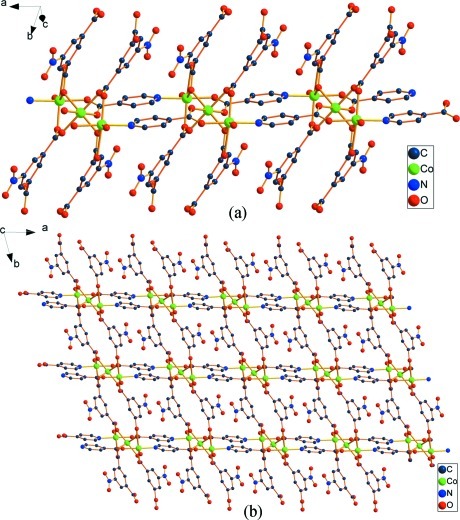
(a) A packing diagram along the a-axial direction. (b) A packing diagram, showing the two-dimensional layer-like structure in the ab plane. H atoms are omitted for clarity.

**Fig. 3.**
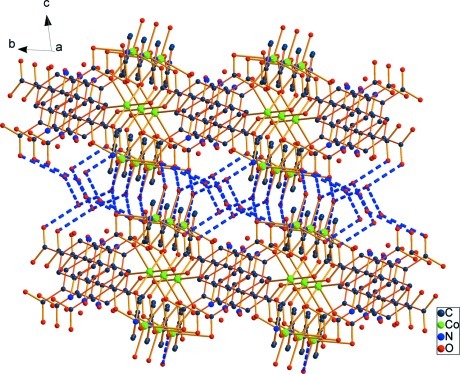
A packing diagram of the title compound, showing the three-dimensional supramolecular network driven by intermolecular hydrogen bonds (dashed lines). H atoms are omitted for clarity.

## supplementary crystallographic information

### Comment

Over the past decades, rational design and construction of metal coordination polymers with aromatic carboxylates have become an attractive area in coordination and supramolecular chemistry, due to the fascinating network topologies they exhibit (Natarajan & Mahata, 2009) and due to industrially focused applications in gas storage, adsorption and separation (Ma *et al.*, 2009), nonlinear optical devices (Zang *et al.*, 2006), and fluorescence (Jiang *et al.*, 2010), *etc*. Due to versatile coordination modes and easy formation of secondary building units, 5-nitroisophthalate (NIPH) has been widely employed to construct coordination polymers (Chen *et al.*, 2010; Du *et al.*, 2008; Sun *et al.*, 2010).

Most cobalt complexes with 5-nitroisophthalate (NIPH) and aromatic N-donor coligands (pyridine, bipyridine, and imidazole) possess low-dimensional structure characteristics, namely, ladder, loop-like chain, zigzag chain, layer, and grid, *etc* (Chen *et al.*, 2006; Du *et al.*, 2008; Luo *et al.*, 2003). Cobalt complexes based on NIPH ligand and other carboxylates have been less developed (Wang *et al.*, 2009). In this work, we selected isonicotinic acid (HIN) as the coligand based on the following considerations: (1) it possesses the bifunctional bridging groups with both oxygen and nitrogen donors as a potential linkers and (2) it can adopt various coordination modes in high-dimensional heterometallic frameworks (Amo-Ochoa *et al.*, 2010). Herein, synthesis and crystal structure of a new cobalt compound is presented, which was prepared by hydrothermal reaction of Co(CH_3_COO)_2_.4 H_2_O with 5-nitroisophthalic acid and isonicotinic acid.

The title compound exhibits a two-dimensional layer-like framework. As illustrated in Fig.1, each asymmetric unit contains one and a half Co^II^ ions, one 5-nitroisophthalate (NIPH) anion, an isonicotinate (IN) anion, one ligated and two lattice water molecules, as the Co1^II^ ion lies on a crystallographic inversion centre. In the structure, the Co1^II^ ion is six-coordinated in an O_6_ donor set with the coordination geometry of a slightly distorted octahedron by four carboxylate oxygen atoms [O1, O1^i^, O4^ii^, O4^iii^, symmetry codes: (i) -*x*, 1 - *y*, 1 - *z*; (ii) *x*, -1 + *y*, *z*; (iii) -*x*, 2 - *y*, 1 - *z*] from four bridging NIPH ligands in the equatorial plane, and the others [O8 and O8^i^, symmetry code: (i) -*x*, 1 - *y*, 1 - *z*] from two IN anions at the axial sites; the Co2^II^ ion coordinates with one N2^iv^ atom [symmetry code: (iv) -1 + *x*, *y*, *z*] and one carboxylate O7 atom from two IN anions, two carboxylate oxygen atoms [O3^ii^ and O7, symmetry code: (ii) *x*, -1 + *y*, *z*] from two different NIPH anions and one ligated water molecules, displaying a distorted square pyramidal coordination geometry. If the weak Co—O interaction between Co2 and O2 (2.4908 (6) Å) was considered, the coordination configuration of the Co2 ion could also be described as a severely distorted octahedron. The Co—O bond distances are in the range of 2.0206 (18)–2.1624 (16) Å, and the Co—N bond length is equal to 2.1465 (19) Å, all of which are in the normal ranges (Du *et al.*, 2008).

The NIPH ligand acts as µ_4_-bridge to link four different Co^II^ ions through two carboxylate groups and the IN anion adopts a tridentate-bridging mode to connect three Co^II^ ions. Thus, one Co1^II^ and two Co2^II^ ions are bridged by six carboxylate groups from four NIPH ligands (two carboxylate groups in bis-monodentate mode and two in monodentate bridged mode) and two IN anions (the carboxylate groups in bis-monodentate mode) to form a linear trinuclear secondary building unit (SBU), [Co_3_(NIPH)_2_(IN)_2_(H_2_O)_2_] with a Co···Co separation of 3.5086 (10) Å. Every trinuclear SBU is in turn linked to its nearest neighbors in the *ab* plane, resulting in a two-dimensional layer like structure perpendicular to the *c*-axis (Fig. 2). Along the *a*-axis direction neighboring molecules are connected through carboxylate and pyridyl units of the IN anions, along the *b*-axis through carboxylate groups of the NIPH ligands. Topologically, the trinuclear Co^II^ units can be seen as four-connected nodes and the organic ligands (the NIPH and IN anions) act as two-connected rods. On the basis of this simplification, this two-dimensional structure can be described as a (4,4)-topological network.

There are multiple O–H···O hydrogen bonds in the complex, which include the following types of contacts: (*a*) between coordinated water molecule and carboxylate oxygen atoms of the NIPH anions [O···O, 2.820 (3) Å], (*b*) between lattice water molecule and carboxylate oxygen atoms of the NIPH anions [O···O, 2.861 (3) – 3.098 (4) Å] and (*c*) between coordinated and lattice water molecules [O···O, 2.648 (3) – 3.297 (6) Å]. Thus, these two-dimensional layers are further extended to a three-dimensional supramolecular network (Fig. 3).

The IR spectrum shows characteristic absorptions for the carboxylate stretching vibrations and the coordination of organic carboxylate anions (NIPH and IN) to Co^II^ was confirmed by the absence of υ(COOH) absorption bands of the organic ligands at around 1700 cm^-1^. The characteristic vibrations of the carboxylate groups are seen in the range 1607–1541 cm^-1^ for asymmetric stretching and 1461–1360 cm^-1^ for symmetric stretching. The broad absorption band observed at 3412 cm^-1^ can be assigned to the O–H stretching vibration, indicating the presence of water molecules.

In summary, a new (4, 4)–two-dimensional layer-like polynuclear Co^II^ coordination polymer was constructed by hydrothermal reaction between Co(II) ions and mixed carboxylate ligands, which is further extended to a three-dimensional supramolecular network through intermolecular hydrogen bonding.

### Experimental

A mixture of Co(CH_3_COO)_2_.4 H_2_O (0.1245 g, 0.50 mmol), 5-nitroisophthalic acid (0.0503 g, 0.25 mmol) and isonicotinic acid (0.0310 g, 0.25 mmol) in 8 ml H_2_O were sealed in a 15 ml Teflon-lined stainless steel reactor and kept under autogenous pressure at 393 K for three days. After the sample was cooled to room temperature at a rate of 5 K/h, pink block-shaped crystals of the title compound were collected and washed with ethanol and water (yield, 45%). IR (KBr pellet, ν, cm^-1^): 3412 (*br*), 3102 (*s*), 1607 (*s*), 1587 (*s*), 1541 (*s*), 1461 (*m*), 1420 (*m*), 1403 (*s*), 1360 (*m*), 1342 (*m*), 1163 (*w*), 1088 (*m*), 1065 (*m*), 1024 (*m*), 933 (*m*), 921 (*w*), 862 (*m*), 792 (*m*), 781 (*m*), 724 (*m*), 714 (*s*), 691 (*m*), 592 (*w*), 562 (*w*).

### Refinement

The disordered hydrogen atoms (H3W and H4W) of lattice water molecule O2W are, due to a close contact of O2W with one of its symmetry equivalent counterparts across an inversion center, disordered in a 1:1 mode in the crystal. In one of the alternative orientations O2W is hydrogen bonding via H3WB to O2W^ii^ and via H4WB to both O2^v^ and O5^vi^. In the other orientation, with O2W acting as the acceptor of a hydrogen bond from O2W^ii^, the water molecule hydrogen bonds via H3WA and H3WB to O6^vi^ and O2^v^ (see Table 1 for symmetry operators). All hydrogen atoms of water molecules were located in electron difference density Fourier maps. The disordered hydrogen atoms (H3W and H4W) of lattice water molecule O2W were refined in a 1:1 disordered mode with O—H distance restraints of 0.85 (2) Å, and H···H distance restraints of 1.37 (2) Å. The other hydrogen atoms were placed geometrically and refined as riding atoms [C—H = 0.93 Å (aromatic C—H), O–H = 0.83 Å (coordinated H_2_O) and 0.85 Å (lattice water molecules)]. All H atoms were refined with isotropic thermal factors *U*_iso_ (H) = 1.2 *U*_eq_ (C), or *U*_iso_ (H) = 1.5 *U*_eq_(O)].

### Crystal data

**Table:**

[Co_3_(C_6_H_4_NO_2_)_2_(C_8_H_3_NO_6_)_2_(H_2_O)_2_]·4H_2_O	*Z* = 1
*M**_r_* = 947.32	*F*(000) = 479
Triclinic, *P*1	*D*_x_ = 1.872 Mg m^−^^3^
Hall symbol: -P 1	Mo *K*α radiation, λ = 0.71073 Å
*a* = 9.1890 (18) Å	Cell parameters from 3665 reflections
*b* = 9.3548 (19) Å	θ = 2.0–27.9°
*c* = 10.390 (2) Å	µ = 1.57 mm^−^^1^
α = 78.74 (3)°	*T* = 298 K
β = 88.64 (3)°	Block, pink
γ = 73.68 (3)°	0.35 × 0.28 × 0.16 mm
*V* = 840.2 (3) Å^3^	

### Data collection

**Table:**

Bruker SMART APEXII CCD area-detector diffractometer	2976 independent reflections
Radiation source: fine-focus sealed tube	2667 reflections with *I* > 2σ(*I*)
Graphite monochromator	*R*_int_ = 0.012
φ and ω scan	θ_max_ = 25.3°, θ_min_ = 2.0°
Absorption correction: multi-scan (*SADABS*; Bruker, 2002)	*h* = −9→11
*T*_min_ = 0.610, *T*_max_ = 0.788	*k* = −11→11
4357 measured reflections	*l* = −12→12

### Refinement

**Table:**

Refinement on *F*^2^	Primary atom site location: structure-invariant direct methods
Least-squares matrix: full	Secondary atom site location: difference Fourier map
*R*[*F*^2^ > 2σ(*F*^2^)] = 0.027	Hydrogen site location: inferred from neighbouring sites
*wR*(*F*^2^) = 0.071	H atoms treated by a mixture of independent and constrained refinement
*S* = 1.02	*w* = 1/[σ^2^(*F*_o_^2^) + (0.0397*P*)^2^ + 0.585*P*] where *P* = (*F*_o_^2^ + 2*F*_c_^2^)/3
2976 reflections	(Δ/σ)_max_ = 0.001
271 parameters	Δρ_max_ = 0.40 e Å^−^^3^
10 restraints	Δρ_min_ = −0.40 e Å^−^^3^

### Special details

**Table:**

Geometry. All e.s.d.'s (except the e.s.d. in the dihedral angle between two l.s. planes) are estimated using the full covariance matrix. The cell e.s.d.'s are taken into account individually in the estimation of e.s.d.'s in distances, angles and torsion angles; correlations between e.s.d.'s in cell parameters are only used when they are defined by crystal symmetry. An approximate (isotropic) treatment of cell e.s.d.'s is used for estimating e.s.d.'s involving l.s. planes.
Refinement. Refinement of *F*^2^ against ALL reflections. The weighted *R*-factor *wR* and goodness of fit *S* are based on *F*^2^, conventional *R*-factors *R* are based on *F*, with *F* set to zero for negative *F*^2^. The threshold expression of *F*^2^ > σ(*F*^2^) is used only for calculating *R*-factors(gt) *etc*. and is not relevant to the choice of reflections for refinement. *R*-factors based on *F*^2^ are statistically about twice as large as those based on *F*, and *R*- factors based on ALL data will be even larger.

### Fractional atomic coordinates and isotropic or equivalent isotropic displacement parameters (Å^2^)

**Table:**

	*x*	*y*	*z*	*U*_iso_*/*U*_eq_	Occ. (<1)
C1	−0.1582 (2)	0.6886 (2)	0.7129 (2)	0.0192 (5)	
C2	−0.1920 (3)	0.8433 (2)	0.6267 (2)	0.0193 (5)	
C3	−0.2761 (3)	0.8738 (2)	0.5103 (2)	0.0215 (5)	
H3	−0.3109	0.7991	0.4846	0.026*	
C4	−0.3067 (3)	1.0182 (3)	0.4336 (2)	0.0226 (5)	
C5	−0.2494 (3)	1.1290 (3)	0.4646 (2)	0.0241 (5)	
H5	−0.2671	1.2232	0.4086	0.029*	
C6	−0.1647 (3)	1.0974 (2)	0.5811 (2)	0.0194 (5)	
C7	−0.1408 (3)	0.9561 (2)	0.6638 (2)	0.0212 (5)	
H7	−0.0902	0.9370	0.7445	0.025*	
C9	0.2058 (2)	0.3927 (2)	0.7504 (2)	0.0201 (5)	
C10	0.3646 (2)	0.3974 (2)	0.7775 (2)	0.0201 (5)	
C11	0.4307 (3)	0.3616 (3)	0.9022 (2)	0.0302 (6)	
H11	0.3769	0.3339	0.9753	0.036*	
C12	0.5780 (3)	0.3674 (3)	0.9166 (2)	0.0291 (5)	
H12	0.6227	0.3388	1.0005	0.035*	
C13	0.5920 (3)	0.4501 (3)	0.6972 (2)	0.0320 (6)	
H13	0.6454	0.4836	0.6259	0.038*	
C14	0.4490 (3)	0.4422 (3)	0.6743 (2)	0.0313 (6)	
H14	0.4085	0.4672	0.5889	0.038*	
C8	−0.1022 (2)	1.2189 (2)	0.6151 (2)	0.0202 (5)	
Co1	0.0000	0.5000	0.5000	0.01527 (11)	
Co2	−0.10352 (3)	0.39808 (3)	0.82492 (3)	0.01738 (10)	
N2	0.6596 (2)	0.4120 (2)	0.81683 (18)	0.0218 (4)	
N1	−0.4063 (2)	1.0548 (2)	0.3153 (2)	0.0297 (5)	
O2	−0.1492 (2)	0.67118 (19)	0.83422 (16)	0.0285 (4)	
O1	−0.14157 (17)	0.57462 (16)	0.65704 (15)	0.0192 (3)	
O6	−0.4433 (3)	0.9517 (2)	0.2817 (2)	0.0474 (5)	
O5	−0.4506 (3)	1.1871 (2)	0.2580 (2)	0.0484 (5)	
O7	0.11919 (18)	0.3888 (2)	0.84536 (16)	0.0285 (4)	
O3	−0.07060 (19)	1.21068 (17)	0.73447 (16)	0.0247 (4)	
O4	−0.08995 (18)	1.31976 (17)	0.52098 (16)	0.0250 (4)	
O8	0.17412 (17)	0.39430 (18)	0.63366 (15)	0.0231 (3)	
O1W	0.8479 (2)	0.0405 (2)	0.1145 (2)	0.0498 (5)	
H1W	0.9262	−0.0299	0.1429	0.075*	
H2W	0.8017	0.0109	0.0599	0.075*	
O3W	−0.0897 (2)	0.2994 (2)	1.01711 (16)	0.0356 (4)	
H5W	−0.0190	0.3181	1.0515	0.053*	
H6W	−0.1021	0.2183	1.0577	0.053*	
O2W	0.3255 (3)	0.0491 (3)	0.0522 (3)	0.0676 (7)	
H4WA	0.271 (8)	0.114 (6)	0.094 (7)	0.101*	0.50
H3WA	0.386 (8)	−0.021 (7)	0.103 (6)	0.101*	0.50
H3WB	0.415 (3)	−0.006 (6)	0.038 (6)	0.101*	0.50
H4WB	0.344 (5)	0.096 (5)	0.114 (5)	0.101*	0.50

### Atomic displacement parameters (Å^2^)

**Table:**

	*U*^11^	*U*^22^	*U*^33^	*U*^12^	*U*^13^	*U*^23^
C1	0.0180 (11)	0.0210 (11)	0.0200 (12)	−0.0090 (9)	−0.0018 (9)	−0.0017 (9)
C2	0.0203 (11)	0.0170 (11)	0.0211 (12)	−0.0057 (9)	0.0012 (9)	−0.0046 (9)
C3	0.0245 (12)	0.0198 (11)	0.0226 (12)	−0.0085 (9)	0.0000 (9)	−0.0067 (9)
C4	0.0246 (12)	0.0229 (12)	0.0205 (12)	−0.0059 (9)	−0.0043 (9)	−0.0050 (9)
C5	0.0285 (13)	0.0181 (11)	0.0244 (13)	−0.0068 (9)	0.0002 (10)	−0.0009 (9)
C6	0.0226 (11)	0.0168 (10)	0.0212 (11)	−0.0087 (9)	0.0018 (9)	−0.0049 (9)
C7	0.0244 (12)	0.0227 (11)	0.0185 (11)	−0.0095 (9)	−0.0006 (9)	−0.0044 (9)
C9	0.0164 (11)	0.0186 (11)	0.0247 (13)	−0.0058 (9)	−0.0022 (9)	−0.0012 (9)
C10	0.0165 (11)	0.0222 (11)	0.0224 (12)	−0.0059 (9)	−0.0030 (9)	−0.0047 (9)
C11	0.0256 (13)	0.0474 (15)	0.0193 (12)	−0.0183 (11)	−0.0009 (10)	0.0010 (11)
C12	0.0247 (13)	0.0447 (15)	0.0191 (12)	−0.0142 (11)	−0.0049 (10)	−0.0017 (11)
C13	0.0258 (13)	0.0518 (16)	0.0201 (13)	−0.0196 (12)	0.0000 (10)	0.0016 (11)
C14	0.0237 (13)	0.0541 (17)	0.0186 (13)	−0.0191 (12)	−0.0043 (10)	−0.0003 (11)
C8	0.0164 (11)	0.0173 (11)	0.0274 (13)	−0.0048 (9)	−0.0008 (9)	−0.0053 (9)
Co1	0.0164 (2)	0.0163 (2)	0.0144 (2)	−0.00812 (16)	−0.00199 (16)	−0.00068 (16)
Co2	0.01635 (17)	0.02053 (17)	0.01603 (17)	−0.00834 (12)	−0.00217 (11)	−0.00047 (12)
N2	0.0185 (10)	0.0277 (10)	0.0205 (10)	−0.0088 (8)	−0.0011 (8)	−0.0042 (8)
N1	0.0334 (12)	0.0289 (11)	0.0256 (11)	−0.0078 (9)	−0.0075 (9)	−0.0031 (9)
O2	0.0386 (10)	0.0315 (9)	0.0176 (9)	−0.0147 (8)	−0.0028 (7)	−0.0024 (7)
O1	0.0226 (8)	0.0158 (7)	0.0209 (8)	−0.0086 (6)	0.0011 (6)	−0.0028 (6)
O6	0.0637 (14)	0.0393 (11)	0.0429 (12)	−0.0196 (10)	−0.0279 (10)	−0.0057 (9)
O5	0.0636 (14)	0.0314 (11)	0.0423 (12)	−0.0081 (10)	−0.0262 (10)	0.0072 (9)
O7	0.0183 (8)	0.0470 (10)	0.0226 (9)	−0.0141 (7)	−0.0003 (7)	−0.0050 (8)
O3	0.0308 (9)	0.0209 (8)	0.0242 (9)	−0.0095 (7)	−0.0051 (7)	−0.0048 (7)
O4	0.0319 (9)	0.0219 (8)	0.0254 (9)	−0.0163 (7)	−0.0005 (7)	−0.0013 (7)
O8	0.0211 (8)	0.0282 (8)	0.0200 (9)	−0.0062 (7)	−0.0051 (6)	−0.0051 (7)
O1W	0.0499 (13)	0.0354 (11)	0.0605 (14)	−0.0130 (10)	−0.0197 (11)	0.0029 (10)
O3W	0.0370 (11)	0.0510 (11)	0.0227 (9)	−0.0284 (9)	−0.0097 (8)	0.0079 (8)
O2W	0.0697 (18)	0.0779 (19)	0.0591 (16)	−0.0224 (14)	−0.0130 (14)	−0.0186 (14)

### Geometric parameters (Å, º)

**Table:**

C1—O2	1.241 (3)	C8—O4	1.246 (3)
C1—O1	1.281 (3)	C8—O3	1.263 (3)
C1—C2	1.499 (3)	Co1—O8	2.0363 (17)
C2—C3	1.388 (3)	Co1—O8^i^	2.0363 (17)
C2—C7	1.389 (3)	Co1—O4^ii^	2.0506 (15)
C3—C4	1.384 (3)	Co1—O4^iii^	2.0506 (15)
C3—H3	0.9300	Co1—O1	2.1623 (16)
C4—C5	1.379 (3)	Co1—O1^i^	2.1623 (16)
C4—N1	1.477 (3)	Co2—O3W	2.0205 (18)
C5—C6	1.391 (3)	Co2—O7	2.0383 (16)
C5—H5	0.9300	Co2—O3^ii^	2.0927 (16)
C6—C7	1.391 (3)	Co2—O1	2.1146 (17)
C6—C8	1.511 (3)	Co2—N2^iv^	2.1468 (19)
C7—H7	0.9300	N2—Co2^v^	2.1468 (19)
C9—O8	1.250 (3)	N1—O6	1.220 (3)
C9—O7	1.254 (3)	N1—O5	1.222 (3)
C9—C10	1.507 (3)	O3—Co2^vi^	2.0927 (16)
C10—C14	1.376 (3)	O4—Co1^vi^	2.0506 (15)
C10—C11	1.384 (3)	O1W—H1W	0.8406
C11—C12	1.383 (3)	O1W—H2W	0.8463
C11—H11	0.9300	O3W—H5W	0.8243
C12—N2	1.334 (3)	O3W—H6W	0.8285
C12—H12	0.9300	O2W—H4WA	0.854 (19)
C13—N2	1.341 (3)	O2W—H3WA	0.83 (2)
C13—C14	1.366 (3)	O2W—H3WB	0.868 (19)
C13—H13	0.9300	O2W—H4WB	0.886 (18)
C14—H14	0.9300		
			
O2—C1—O1	120.9 (2)	O8—Co1—O4^iii^	84.51 (7)
O2—C1—C2	121.4 (2)	O8^i^—Co1—O4^iii^	95.49 (7)
O1—C1—C2	117.69 (19)	O4^ii^—Co1—O4^iii^	180.0
C3—C2—C7	120.2 (2)	O8—Co1—O1	89.18 (6)
C3—C2—C1	119.64 (19)	O8^i^—Co1—O1	90.82 (6)
C7—C2—C1	120.2 (2)	O4^ii^—Co1—O1	87.91 (6)
C4—C3—C2	118.2 (2)	O4^iii^—Co1—O1	92.09 (6)
C4—C3—H3	120.9	O8—Co1—O1^i^	90.82 (6)
C2—C3—H3	120.9	O8^i^—Co1—O1^i^	89.18 (6)
C5—C4—C3	122.5 (2)	O4^ii^—Co1—O1^i^	92.09 (6)
C5—C4—N1	119.0 (2)	O4^iii^—Co1—O1^i^	87.91 (6)
C3—C4—N1	118.4 (2)	O1—Co1—O1^i^	180.0
C4—C5—C6	118.8 (2)	O3W—Co2—O7	86.46 (7)
C4—C5—H5	120.6	O3W—Co2—O3^ii^	101.78 (7)
C6—C5—H5	120.6	O7—Co2—O3^ii^	97.35 (7)
C5—C6—C7	119.6 (2)	O3W—Co2—O1	158.16 (7)
C5—C6—C8	118.8 (2)	O7—Co2—O1	93.45 (7)
C7—C6—C8	121.6 (2)	O3^ii^—Co2—O1	99.89 (6)
C2—C7—C6	120.5 (2)	O3W—Co2—N2^iv^	90.42 (8)
C2—C7—H7	119.8	O7—Co2—N2^iv^	175.95 (7)
C6—C7—H7	119.8	O3^ii^—Co2—N2^iv^	85.80 (7)
O8—C9—O7	126.8 (2)	O1—Co2—N2^iv^	88.50 (7)
O8—C9—C10	115.7 (2)	C12—N2—C13	116.3 (2)
O7—C9—C10	117.6 (2)	C12—N2—Co2^v^	126.40 (16)
C14—C10—C11	117.4 (2)	C13—N2—Co2^v^	116.62 (15)
C14—C10—C9	119.2 (2)	O6—N1—O5	123.4 (2)
C11—C10—C9	123.4 (2)	O6—N1—C4	118.2 (2)
C12—C11—C10	119.0 (2)	O5—N1—C4	118.4 (2)
C12—C11—H11	120.5	C1—O1—Co2	99.61 (13)
C10—C11—H11	120.5	C1—O1—Co1	132.30 (13)
N2—C12—C11	123.7 (2)	Co2—O1—Co1	110.24 (7)
N2—C12—H12	118.2	C9—O7—Co2	122.97 (15)
C11—C12—H12	118.2	C8—O3—Co2^vi^	124.57 (14)
N2—C13—C14	123.5 (2)	C8—O4—Co1^vi^	134.35 (15)
N2—C13—H13	118.3	C9—O8—Co1	138.09 (15)
C14—C13—H13	118.3	H1W—O1W—H2W	108.3
C13—C14—C10	120.1 (2)	Co2—O3W—H5W	107.7
C13—C14—H14	120.0	Co2—O3W—H6W	133.6
C10—C14—H14	120.0	H5W—O3W—H6W	109.9
O4—C8—O3	126.6 (2)	H4WA—O2W—H3WA	111 (4)
O4—C8—C6	115.6 (2)	H4WA—O2W—H3WB	149 (7)
O3—C8—C6	117.70 (19)	H3WA—O2W—H3WB	51 (7)
O8—Co1—O8^i^	180.000 (1)	H4WA—O2W—H4WB	46 (6)
O8—Co1—O4^ii^	95.49 (7)	H3WA—O2W—H4WB	77 (6)
O8^i^—Co1—O4^ii^	84.51 (7)	H3WB—O2W—H4WB	103 (3)
			
O2—C1—C2—C3	−146.5 (2)	C3—C4—N1—O5	169.9 (2)
O1—C1—C2—C3	32.3 (3)	O2—C1—O1—Co2	0.1 (2)
O2—C1—C2—C7	32.7 (3)	C2—C1—O1—Co2	−178.70 (16)
O1—C1—C2—C7	−148.5 (2)	O2—C1—O1—Co1	−128.83 (19)
C7—C2—C3—C4	0.0 (3)	C2—C1—O1—Co1	52.4 (3)
C1—C2—C3—C4	179.3 (2)	O3W—Co2—O1—C1	8.5 (2)
C2—C3—C4—C5	4.0 (3)	O7—Co2—O1—C1	−80.54 (13)
C2—C3—C4—N1	−175.2 (2)	O3^ii^—Co2—O1—C1	−178.62 (13)
C3—C4—C5—C6	−3.7 (4)	N2^iv^—Co2—O1—C1	95.91 (13)
N1—C4—C5—C6	175.4 (2)	O3W—Co2—O1—Co1	150.72 (15)
C4—C5—C6—C7	−0.5 (3)	O7—Co2—O1—Co1	61.64 (8)
C4—C5—C6—C8	−179.9 (2)	O3^ii^—Co2—O1—Co1	−36.44 (8)
C3—C2—C7—C6	−4.1 (3)	N2^iv^—Co2—O1—Co1	−121.91 (8)
C1—C2—C7—C6	176.6 (2)	O8—Co1—O1—C1	84.14 (19)
C5—C6—C7—C2	4.4 (3)	O8^i^—Co1—O1—C1	−95.86 (19)
C8—C6—C7—C2	−176.2 (2)	O4^ii^—Co1—O1—C1	179.66 (19)
O8—C9—C10—C14	16.8 (3)	O4^iii^—Co1—O1—C1	−0.34 (19)
O7—C9—C10—C14	−162.7 (2)	O8—Co1—O1—Co2	−41.04 (7)
O8—C9—C10—C11	−164.6 (2)	O8^i^—Co1—O1—Co2	138.96 (7)
O7—C9—C10—C11	16.0 (3)	O4^ii^—Co1—O1—Co2	54.48 (8)
C14—C10—C11—C12	−2.2 (4)	O4^iii^—Co1—O1—Co2	−125.52 (8)
C9—C10—C11—C12	179.2 (2)	O8—C9—O7—Co2	−4.1 (3)
C10—C11—C12—N2	2.8 (4)	C10—C9—O7—Co2	175.29 (14)
N2—C13—C14—C10	1.9 (4)	O3W—Co2—O7—C9	157.95 (19)
C11—C10—C14—C13	0.0 (4)	O3^ii^—Co2—O7—C9	56.52 (18)
C9—C10—C14—C13	178.7 (2)	O1—Co2—O7—C9	−43.92 (18)
C5—C6—C8—O4	−21.3 (3)	O4—C8—O3—Co2^vi^	35.3 (3)
C7—C6—C8—O4	159.2 (2)	C6—C8—O3—Co2^vi^	−143.13 (16)
C5—C6—C8—O3	157.3 (2)	O3—C8—O4—Co1^vi^	7.4 (4)
C7—C6—C8—O3	−22.2 (3)	C6—C8—O4—Co1^vi^	−174.15 (14)
C11—C12—N2—C13	−1.0 (4)	O7—C9—O8—Co1	43.6 (4)
C11—C12—N2—Co2^v^	−171.6 (2)	C10—C9—O8—Co1	−135.76 (18)
C14—C13—N2—C12	−1.3 (4)	O4^ii^—Co1—O8—C9	−101.0 (2)
C14—C13—N2—Co2^v^	170.1 (2)	O4^iii^—Co1—O8—C9	79.0 (2)
C5—C4—N1—O6	172.6 (2)	O1—Co1—O8—C9	−13.2 (2)
C3—C4—N1—O6	−8.3 (3)	O1^i^—Co1—O8—C9	166.8 (2)
C5—C4—N1—O5	−9.3 (3)		

Symmetry codes: (i) −*x*, −*y*+1, −*z*+1; (ii) *x*, *y*−1, *z*; (iii) −*x*, −*y*+2, −*z*+1; (iv) *x*−1, *y*, *z*; (v) *x*+1, *y*, *z*; (vi) *x*, *y*+1, *z*.

### Hydrogen-bond geometry (Å, º)

**Table:**

*D*—H···*A*	*D*—H	H···*A*	*D*···*A*	*D*—H···*A*
O1*W*—H1*W*···O3^vii^	0.84	2.05	2.861 (3)	160
O1*W*—H2*W*···O2*W*^viii^	0.85	1.93	2.773 (3)	173
O3*W*—H5*W*···O2^ix^	0.82	2.01	2.820 (2)	168
O3*W*—H6*W*···O1*W*^x^	0.83	1.83	2.648 (3)	167
O2*W*—H4*WA*···O2^i^	0.85 (2)	2.27 (2)	3.098 (4)	165 (8)
O2*W*—H3*WA*···O6^xi^	0.83 (2)	2.38 (6)	3.055 (3)	138 (8)
O2*W*—H3*WB*···O2*W*^viii^	0.87 (2)	2.49 (3)	3.297 (6)	154 (6)
O2*W*—H4*WB*···O6^xi^	0.89 (5)	2.54 (5)	3.055 (4)	118 (3)
O2*W*—H4*WB*···O2^i^	0.89 (5)	2.54 (5)	3.098 (3)	121 (4)

Symmetry codes: (i) −*x*, −*y*+1, −*z*+1; (vii) −*x*+1, −*y*+1, −*z*+1; (viii) −*x*+1, −*y*, −*z*; (ix) −*x*, −*y*+1, −*z*+2; (x) *x*−1, *y*, *z*+1; (xi) *x*+1, *y*−1, *z*.
